# Protective Effects of Novel *Lactobacillaceae* Strains Isolated from Chicken Caeca against Necrotic Enteritis Infection: *In Vitro* and *In Vivo* Evidences

**DOI:** 10.3390/microorganisms10010152

**Published:** 2022-01-12

**Authors:** Nuria Vieco-Saiz, Yanath Belguesmia, Ruth Raspoet, Eric Auclair, Connor Padgett, Christopher Bailey, Frédérique Gancel, Djamel Drider

**Affiliations:** 1 UMR Transfrontalière BioEcoAgro1158, Univ. Lille, INRAE, Univ. Liège, UPJV, YNCREA, Univ. Artois, Univ. Littoral Côte d’Opale, ICV—Institut Charles Viollette, F-59000 Lille, France; nuriavieco@gmail.com (N.V.-S.); yanath.belguesmia@univ-lille.fr (Y.B.); frederique.gancel@univ-lille.fr (F.G.); 2 Phileo Lesaffre Animal Care, 137 Rue Gabriel Péri, F-59700 Marcq-en-Barœul, France; r.raspoet@phileo.lesaffre.com (R.R.); e.auclair@phileo.lesaffre.com (E.A.); 3 Phileo by Lesaffre, 7475 W Main St., Milwaukee, WI 53214, USA; c.padgett@phileo.lesaffre.com; 4 Department of Poultry Science, Texas A&M University, 101 Kleberg Center, 2472 TAMU, College Station, TX 77845, USA; c-bailey@tamu.edu

**Keywords:** *Limosilactobacillus reuteri*, *Ligilactobacillus salivarius*, probiotics, cytotoxicity, biofilm, *Clostridium perfringens*, necrotic enteritis (NE), *in vivo*, chicken gastrointestinal tract

## Abstract

The present study aimed to show the benefits of novel lactic acid bacteria (LAB) strains isolated from the caeca of healthy chickens. These novel strains, identified as *Limosilactobacillus reuteri* and *Ligilactobacillus salivarius*, displayed high levels of lactic acid production, capability of biofilm formation, high aggregation and adhesion scores, and significant survival rates under conditions mimicking the chicken gastrointestinal tract (GIT). In addition, these novel *Lactobacillaceae* isolates were neither hemolytic nor cytotoxic. *In vivo* trials were able to establish their ability to reduce necrotic enteritis. Notably, a significant weight gain was registered, on day 10 of treatment, in the group of chickens fed with a mixture of *L.* *reuteri* ICVB416 and *L.* *salivarius* ICVB430 strains, as compared with the control group. This group has also shown a reduced number of lesions in the gut compared with other infected chicken groups. This study provides *in vitro* and *in vivo* evidence supporting the benefits of these novel *Lactobacillaceae* isolates for their use in poultry livestock as protective cultures to control the bacterial necrotic enteritis (NE) *Clostridium perfringens*.

## 1. Introduction

The use of antibiotics as growth promoters in industrial livestock farming remains a controversial issue. Countries from the European Union (Regulation 1831/2003/EC), as well as Mexico, New Zealand and South Korea, have adopted since 2006 laws banning the use of antibiotics as a preventive measure in livestock farming [1]. The reasons for such a decision were mainly associated with the development of antimicrobial resistance (AMR), a serious global health concern. 

Currently, around 700,000 people die each year in the world because of AMR-associated diseases [2]. In the USA, at least 2.8 million people suffer an antibiotic resistant infection, of which 35,000 die [3], and the EU counterpart is estimated at 33,000 annually [4]. The AMR crisis needs immediate actions according to different official sources. If left unaddressed, AMR could force 24 million people into extreme poverty by 2030, and will cause 10 million deaths per year around the world with a cumulative cost of US $100 trillion by 2050 [2,5,6].

Therefore, international organizations like WHO, FAO and OIE, but also scientists, and governments are encouraging the development of alternative approaches for replacing aging antibiotics and tackling the AMR phenomenon. In light of this, a holistic approach called One Health was proposed with the aim of rationalizing the usage of antibiotics in humans and animals, considering environmental constraints and the needs of the food industry [7]. *Clostridium perfringens* type G is responsible for a severe infection in chickens known as bacterial necrotic enteritis (NE). This detrimental infection was shown to be associated with the production of toxins, mainly NetB [8,9], which acts by osmotic cell lysis on enterocytes, causing their death [9,10]. It should be noted that NetB toxin is likely the main virulence factor associated with the avian NE. *Clostridium* strains are classified into A to G biotypes, based on the toxin produced. Thus, strains producing NetB and α-toxin are included in the newly created biotype G [9]. The α-toxin is a zinc metalloenzyme endowed with hemolytic and dermonecrotic activities [11]. This toxin is responsible for extensive plasma membrane degradation accompanied by a release of lactate dehydrogenase that characterizes necrosis [10]. It is also worth noting that NE is induced by other factors, including coccidiosis, mycotoxins, immunosuppressive agents, nutritional factors and anthropogenic factors of farm management [12]. The bacterial NE infection is responsible for major economic damages in the poultry industry [13]. 

To tackle AMR, different therapeutic options were suggested, including the use of prebiotics, probiotics from fungal and bacterial sources, bacteriophages, antimicrobial peptides (AMPs), antibodies, vaccines, organic acids and enzymes [14,15].

Probiotics are defined by FAO/WHO as living microorganisms able to provide benefits to the host health when adequately and sufficiently administered [16]. Lactic acid bacteria (LAB) are part of the normal chicken gut microbiota, and play a key role in the maintenance of gut homeostasis, as well as in the control of pathogens through the production of different antimicrobials including bacteriocins, ethanol and CO_2_ [17]. In addition to these antimicrobials, LAB may help to inhibit the invasion of pathogens through complex competitive exclusion mechanisms [18,19,20]. Some of them can modulate the host immune system by affecting the innate and adaptive immune responses, and induce the production of cytokines [21]. Notably, LAB can increase the bioavailability of nutrients such as fatty acids and vitamins, improve the digestibility of proteins, fiber and phosphorus, which are then mostly absorbed by the host [22,23,24]. LAB strains also have capabilities that can stimulate the production of mucus by the intestinal cells [25,26,27]. Some LAB species, such as *Limosilactobacillus reuteri* and *Ligilactobacillus salivarius*, are commonly found in the chicken gut microbiota. These species are reported as adequate probiotic candidates to improve zootechnical performance and poultry health [28].

In this study, we characterized *in vitro* the potential of newly isolated strains viz *L. reuteri* ICV416, *L. salivarius* ICV421 and *L. salivarius* ICV430, and then established *in vivo* their capability to control NE, which is a severe infection caused by *Clostridium perfringens*. 

## 2. Material and Methods

### 2.1. Isolation of Lactic Acid Bacteria from Chicken Caeca and Their Mass Spectrometry Identification

The bacterial strains were isolated from the caeca of three different chickens (Ross 308) raised in a local farm located in the North of France. LAMVAB media, which corresponds to the de Man Rogosa and Sharpe medium supplemented with L-cysteine-HCl (0.25 g/L) (Sigma-Aldrich, St Louis, MO, USA) and vancomycin (20 mg/L) [29], was used for the isolation of the LAB strains. Therefore, isolates grown on this medium, displaying a Gram-positive staining, devoid of catalase and oxidase activities, were presumably considered as LAB. These isolates were then identified by MALDI-TOF mass spectrometry (Autoflex speed, Bruker Daltonics, Bremen, Germany) performed as described by Zidour et al. [30]. The RAPD-PCR approach using M13 primer [5′-GAGGGTGGCGGTTCT-3′] [31] was performed, enabling the discarding of the redundant LAB strains. 

### 2.2. Anti-Clostridium perfringens Activity 

The 49 LAB strains isolated in this work were tested against *C. perfringens* DSM 756 as well as against six other *Clostridium* strains isolated from sick chickens, including *C. perfringens* ICVB081: a strain harboring the *netB* gene [32]. Two methods were used to assess the anti-*C. perfringens* activity: the slab test using the whole bacteria and their metabolites according to the protocol described by Dec et al. [33]; and the wells test using the neutralized supernatant according to Bendali et al. [34]. These protocols are detailed thereafter. 

*Clostridium* strains grown under anaerobic conditions were plated onto brain heart infusion (BHI) agar medium (1% (*w/v*) agar) by swabbing. 

LAB strains were cultured in 5 mL of MRS broth at 37 °C for 20 h. The resulting culture was centrifuged (10,000× *g*, 10 min, 4 °C), and the culture supernatant was separated from the pellets. 

For the slab test, the culture was adjusted to an OD_600_ of 0.4 with phosphate-buffered saline (PBS). Then, 200 μL were spread on a zone of ~4 cm diameter on Petri plates containing MRS agar. After incubation for 20 h at 37 °C, a 6 mm diameter agar column with all of its surface grown with LAB (slab) was cut and placed on top of the agar BHI already inoculated with *C. perfringens*. 

The wells test was performed according to Bendali et al. [34]. The culture was centrifuged (10,000× *g*, 10 min, 4 °C), and the cell-free culture supernatant was recovered. The pH was adjusted to 6.30–6.60 using 3 M sodium hydroxide (NaOH) and then filtered through a polyethersulfone membrane of 0.45 μm porosity (Corning, Corning, NY, USA). A volume of 40 μL of the neutralized supernatant was deposited in the wells formed purposefully on the Petri plates containing the requested growth medium. 

For both assays, the plates were incubated at 4 °C for 2 h in order to allow diffusion of the putative antibacterial substances. After incubation at 37 °C for 20 h under anaerobic conditions, the plates were inspected for development of zones of inhibition, and in case of a positive result, the radials were measured. *Enterococcus faecalis* 14 producing a leaderless class IIb enterocin 14 (Ent DD14), known for its activity against *C. perfringens* [35], was used as a positive control. 

### 2.3. Quantification of Lactic Acid Production 

The quantity of lactic acid produced by these newly isolated LAB strains was determined by high performance liquid chromatography (HPLC) assay after a standard culture in MRS medium for 20 h at 37 °C. An optical density (OD) reading at 600 nm and pH measurement were performed and followed by centrifugation (10,000× *g*, 10 min, 4 °C) to recover the cell-free culture supernatants, which were filtered through Millipore filters (0.2 μm) (Burlington, MA, USA). Lactic acid was quantified by the HPLC Spectra System P1000 XR (Thermo Fisher, Waltham, MA, USA). The column used was a Fast Juice Column (50 mm × 7.8 mm, Phenomenex, Torrance, CA, USA) with isocratic elution with H_3_PO_4_ (0.05 % *w/w*), at a flow rate of 0.8 mL/min and a temperature of 55 °C. 

### 2.4. Biofilm Formation

Bacterial cultures, 18 h in age, recovered at an OD_600nm_ of 5 and were diluted at 1:50 using fresh MRS broth in 96-well polystyrene plates. Once inoculated, these plates were anaerobically incubated at 37 °C for 24 h. The protocol described by Jones and Versalovic [36] was used to quantify the formed biofilms. To be brief about the culture time, the plates were washed twice to remove the non-adherent bacteria from the bottom of the wells. A solution of 0.1% (*w/v*) crystal violet (Sigma-Aldrich) was used to stain the bacteria forming biofilms. After 15 min of incubation at 37 °C and centrifugation (200 rpm), the plates were washed again with distilled water. Finally, 96% (*v/v*) of ethanol was added to remove the leftover crystal violet. The absorbance was recorded at 600 nm using a Safas MP96 microplate reader (Safas, Monaco, France) in order to quantify the biofilm formation. 

### 2.5. Resistance to Gastrointestinal Conditions in Chicken 

Conditions mimicking the different stages of the digestion in the crop, gizzard and intestine of chicken were studied. For the crop, the following conditions were applied: pH of 4.5 for 45 min, followed by a decrease of pH to 3.5 for 90 min in the presence of pepsin (3 mg/mL) (Sigma-Aldrich) and 2 g of glass beads (2 mm in diameter), shaken at 350 rpm to simulate grinding in the gizzard. Finally, an increase of pH to 6.4 and an addition of pancreatin at 1 mg/mL (Sigma-Aldrich) and bile oxgall at 0.35% (Sigma-Aldrich) were applied for 3 h to mimic the passage through the intestine [37,38]. All of these steps were performed at 41.3 °C, which corresponds to the physiological temperature of the chicken [39]. *Lacticaseibacillus rhamnosus* ATCC 7469 and *E. faecalis* 14 were used as controls [35,40]. Strain cultures, 20 h in age and grown at 37 °C, were centrifuged (5000× *g*, 15 min, 4 °C), and the resulting pellets were recovered, washed with 0.9 % (*w/v*) of sodium chloride (NaCl) and resuspended in different solutions corresponding to the conditions described above. Samples were taken and bacterial cells were counted at T = 0 after each step/compartment and cultivation on MRS agar plates. The number of bacteria and the strain viability were determined by flow cytometry (Attune NxT, Thermo Fisher, Waltham, MA, USA). The dyes used to assess LAB viability were syto-24 (Invitrogen, Waltham, MA, USA) and propidium iodide (Molecular Probes, Eugene, OR, USA) at final concentrations of 10 nM and 200 nM, respectively. Flow cytometer channels were set up as follows: FSC 100, SSC 300. After data acquisition, a common gate was determined to follow the changes in bacteria viability in all of the gastrointestinal steps.

### 2.6. Adhesion to Intestinal Cells

Adhesion of *C. perfringens* and newly isolated LAB strains on Caco-2 intestinal epithelial cells was determined *in vitro*. To this end, Caco-2 cells were seeded in 24-well plates, at a loading of 4 × 10^4^ cells per well, and incubated for 7 days at 37 °C in a 95% humidity with 5% CO_2_ in Dulbecco’s modified eagle medium (DMEM) (Lonza, Bâle, Switzerland) supplemented with 25 mM glucose, 5 mM L-glutamine, 10 % (*v/v*) fetal calf serum (FBS) and 100 U/mL penicillin and streptomycin. 

For the bacterial adhesion test, cultures of LAB and *C. perfringens* strains were prepared in MRS and BHI broth, respectively. The bacterial cells were recovered by centrifugation (8000× *g*, 10 min, 4 °C), washed twice with PBS buffer and finally resuspended in a non-complemented DMEM medium. Then, a volume of 500 µL of these suspensions was added to each well containing Caco-2 cells at a ratio of 1:10 (Caco-2 cell: bacterial cells). After incubation at 37 °C for 2 h, Caco-2 cell monolayers were washed twice with PBS at 37 °C, treated with 200 µL trypsin/EDTA (Gibco, Waltham, MA, USA) and incubated for 10 min at 37 °C to detach the cells. The percentage of adhesion was determined via the method described by Candela et al. [41] using real-time PCR. 

### 2.7. qPCR Assay

An aliquot of 20 μL of each sample was transferred to a 0.2 mL tube and incubated for 10 min at room temperature with 3.8 μL of trypsin inhibitor solution (Sigma-Aldrich). The real-time quantitative PCR (QuantStudio^®^3, Applied Biosystems, Waltham, MA, USA) was performed with the fluorophore SYBR Green I (Molecular Probes) in order to quantify the fluorescent signal. The primers used here were CPerf165F [5′-CGCATAACGTTGAAAGATGG-3′] and CPerf269R [5′-CCTTGGTAGGCCGTTACCC-3′] for *C. perfringens* [42], and LactoG1F [5′-TGGAAACAGRTGCTAATACCG-3′] and LactoG1R [5′-GTCCATTGTGGAAGATTCCC-3′] for *Lactobacillaceae*.

Amplification was performed in a final volume of 20 μL containing 2 μL of the cell suspension, 0.4 μM of each primer and supplemented with SYBR^®^ Select Master Mix (Applied Biosystems). The program applied was the following: pre-incubation step at 94°C for 10 min, 40 cycles of amplification (95 °C for 15 s, 60 °C for 10 s, 72 °C for 30 s) and finally 72 °C for 10 min. For absolute quantification, dilutions of each tested bacterium in PBS with concentrations of 10^3^ to 10^6^ CFU/mL were used as standards.

### 2.8. Biosafety Aspects of Newly Selected LAB Strains

#### 2.8.1. Hemolytic Activity

LAB strains were pre-cultured in MRS broth at 37 °C for 24 h. After isolation on MRS agar, three colonies were randomly selected and plated onto a Columbia agar + horse blood (Oxoid, Basingstoke, UK) medium. The plates were incubated anaerobically at 37 °C and were inspected for any hemolysis after 24, 48 and 72 h. The presence of a clear area converges for a complete hemolysis (Β-hemolysis), whilst the greenish hemolysis (α-hemolysis) is known as an incomplete hemolysis. The absence of any hemolysis (γ-hemolysis) is usually observed for *Lactobacillaeae* [43]. Here, *Staphylococcus aureus* ATCC 25923 (Β-hemolytic) was used as a positive control strain [44]. 

#### 2.8.2. Antibiotic Resistance

The bacterial cultures were swabbed uniformly onto the surface of the agar medium. After drying, antibiotic discs were applied, and the plates were incubated at 37 °C for a period of 24–48 h. After this period of incubation, the plates were carefully examined and the radials of the zones of inhibition were recorded. Sensibility and resistance traits of these novel LAB strains were determined according to the recommendations of the EUCAST manual [45]. The genome of the selected LAB strains was analyzed for the presence of antibiotic resistance genetic determinants. DNA of each strain was extracted and sequenced using the protocol described by Al Seraih et al., [46]. Genome annotation was performed using RAST (http://rast.nmpdr.org, accessed on 15 May 2018).

#### 2.8.3. Cytotoxicity

HT29 cells were grown at 37 °C in a humid atmosphere with 5% CO_2_ in DMEM medium (Lonza) supplemented with 25 mM glucose, 5 mM L-glutamine, 10% (*v*/*v*) fetal calf serum (FBS), 100 U/mL penicillin and streptomycin. The test was performed with 30,000 HT29 cells. 

The selected LAB strains were grown for 36 h at 37 °C in MRS broth. Cultures were centrifuged (10 min, 10,000× *g*, 4 °C) and resuspended in DMEM without antibiotics or FBS. Contact was made for 24 h at 37 °C and 5% CO_2_. Bacteria were tested at 10^5^ CFU/well, or 10^7^ CFU/well. Heat-inactivated bacteria (95 °C for 5 min) were used as a control to ascertain that no cytotoxic components were present on the bacterial surface.

To measure the cytotoxic effect, after contact, the cells were washed twice with PBS in order to remove bacteria. DMEM medium, which is supplemented with gentamicin (50 μg/mL) and 5 % of the CCK-8 “Cell Counting Kit-8” reagent (Dojindo Molecular Technology, Rockville MD, USA), was added to each well. The OD at 480 nm was measured after 2 h. Notably, these tests were performed in triplicate.

### 2.9. Coccidiostat Analysis

Sensitivity to coccidiostat was determined by using the minimum inhibitory concentration (MIC) based on the method of CLSI M45 [47]. Strains were grown overnight at 37 °C in LAB susceptibility test medium (LSM) under anaerobic conditions. Coccidiostats solutions (monensin sodium salt, narasin, salinomycin, maduramicin ammonium, lasalocid A sodium salt, decoquinate, diclazuril, halofuginone hydrobromide, robenidine hydrochloride, narasin:nicarbazin) were freshly prepared on the day of testing in DMSO at concentrations suggested by the EFSA [48]. Notably, DMSO was used at 5% (*v/v*), at which concentration DMSO has no effect on cell viability (data not shown). A volume of 10 µL of bacterial suspension (5 × 10^6^ cells/mL) was inoculated in the wells with coccidiostat dilutions that were prepared in 100 µL of LSM medium. The microplates were incubated and the OD_600_ was read at 48 h. *S. aureus* ATCC29213 grown in BHI for 24 h in aerobic conditions was used as a control [49,50]. 

### 2.10. In Vivo Evaluation of the Three Newly Isolated Lactobacillaceae Strains 

A trial was conducted to determine the efficacy of these newly isolated LAB strains and evaluate their potential application as preventive agents to control NE in broiler chickens from a cross of the Cobb 500 (female) and Hubbard M99 (male) lines. This study was performed using *L. reuteri* ICV416, *L. salivarius* ICV421 and *L. salivarius* ICV430 strains regarding their promising probiotic features assessed *in vitro*. Notably, *in vivo* experiments were conducted for 17 days on 240 chickens (30 animals split in 6 cages per treatment group). The trial started on the day of hatching and set up as a near complete factorial design. During trials, LAB strains were administered alone (*L. reuteri* ICVB416, *L. salivarius* ICVB421 or *L. salivarius* ICVB430), in pairwise combination (*L. reuteri* ICVB416 and *L. salivarius* ICVB421; *L. reuteri* ICVB 416 and *L. salivarius* ICVB 430) and all three together, knowing that no potential cross-killing effect exists (data not shown). These strains were administered by oral gavage once daily, on days 1 and 2, and then again from day 10 to day 13. 

#### 2.10.1. Chicken Environment and Diet

The chickens were raised in cages with an average of 432 cm^2^ per chicken. The cages were placed at several levels in an air-conditioned room, maintained at room temperature throughout the study and cleared for testing with a level 2 biohazard. The conditions were selected according to the Guide for the Care and Use of Agricultural Animals in Research and Education (https://www.aaalac.org/about/ag_guide_3rd_ed.pdf, accessed on 3 June 2018). After the approval of the Institutional Animal Care and Use Committee (IACUC), under the number 2018–009 at the United States Department of Agriculture—Agricultural Research Service (College Station, TX, USA), the test was started. Lighting was provided 24 h a day for the duration of the study. The birds were given water and ad-libitum food. The diet consisted of the mash feed underlie of corn and soy for a starter until day 9. Then, from day 10, the grower diet included wheat. The diet’s compositions are listed in Tables S1 and S2 (Supplementary Materials). 

#### 2.10.2. Vaccination

Except for the non-challenged group, the chickens were vaccinated on day 1 with coccidiosis vaccine Advent (Huvepharma, Peachtree, GA USA) at a high dose (9×) to challenge birds. Advent vaccine contains low pathogenic, highly immunogenic and coccidiostat-sensitive strains of *Eimeria maxima*, *E. tenella* and *E. acervulina*. On day 9, the chickens were vaccinated intraocularly against avian infectious bursal disease with Intervet, Bursal Vac-G603.

#### 2.10.3. *C. perfringens* Infection Protocol

*C. perfringens*–containing medium was provided by the ARS, Southern Plains Agricultural Research Center, USDA. The isolation and preparation of *C. perfringens* were as described by McReynolds et al. [51]. The *C. perfringens* pathogen was a combination of four formerly type A field isolates from three different regions (Georgia, Texas and Virginia). The wild type *C. perfringens* strains were grown overnight at 37 °C in thioglycollate broth as previously described [52]. After this, it was administered at a concentration of 10^7^ CFU/mL with a volume of 3 mL by oral gavage dose in a sterile thioglycollate, using a 20-gauge dosing needle at 14–16 days of age. 

#### 2.10.4. Administration of LAB Strains 

LAB strains were grown overnight at 37 °C in MRS. Cell pellets were recovered and resuspended in PBS, and 250–500 µL of obtained LAB strain suspension, at 10^7^ CFU/mL, were administered orally to the birds on days 1 and 2. Notably, when the birds were 10–13 days old, the dosage was increased to 1 mL, but still at a concentration of 10^7^ CFU/mL. The control groups (NCp and Cp) were treated in a similar way and received the same volumes of sterile PBS solution. The birds used for the negative control were treated first to reduce the risk of cross-contamination.

#### 2.10.5. Measurements of Feed Uptake and Weight of Birds

The study ended on day 17. Bird performance was measured on days 0, 10, 14 and 17 of the experimental periods by recording bird weight (in g) and feed uptake for each cage. This made it possible calculate the “Feed Conversion Ratio”, which is defined as the feed consumed in kg per kg of body weight gain of the animal. This is an important measure to evaluate the economic performance of the farm [53]. On day 10, one chicken from each pen unit was necropsied, and sampling was done. At the end of the trial, all of the remaining birds were euthanized by CO_2_ asphyxiation. NE lesions were scored in the intestines and analyzed following the recommendations of Prescott et al. [54].

### 2.11. Statistical Analysis 

*In vitro* studies were performed in triplicate. *In vivo* assay had five pen replicates for each treatment. Statistical comparisons between the different results obtained were made by analysis of variance ANOVA using Statgraphics^®^ Centurion XVI software (Statpoint Technologies, Warrenton, VA, USA).

## 3. Results

### 3.1. Diversity of LAB Strain in Chicken Ceca

The 70 isolates obtained from the caeca of chickens using LAMVAB medium were identified by MALDI-TOF-MS and characterized by RAPD-PCR to discard duplicates and redundant LAB strains. This procedure made it possible to identify 28 *L. salivarius* strains, 14 *L. reuteri*, 1 *Lactobacillus gallinarum*, 1 *Lactobacillus gasseri*, 1 *Lactobacillus johnsonii*, 1 *Limosilactobacillus antri*, two *Streptococcus lutetiensis* and 2 *Streptococcus alactolyticus*.

### 3.2. Newly ISOLATED LAB Strains Displayed Strong Anti-Clostridium perfringens Activity

**Figure 1:**
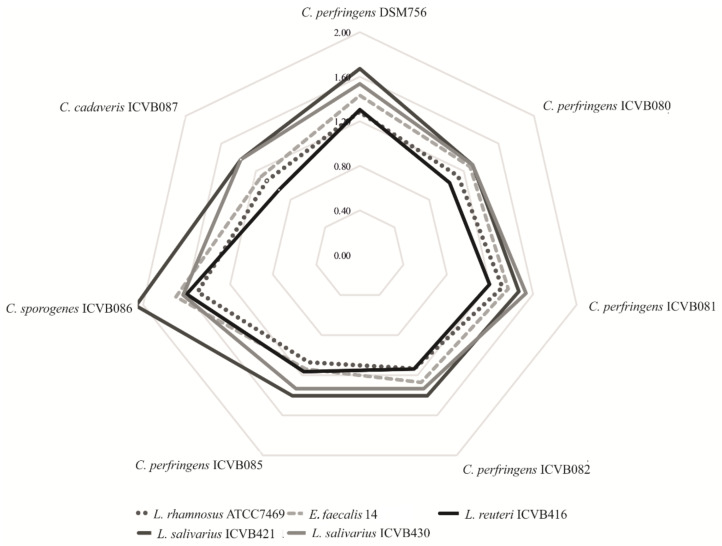
Anti-*Clostridium* activity depicted as a spider chart. Zones of inhibition were assessed by measuring the *radius* of the halo in cm. Target strains are in the borders and lines show the activity of probiotic candidates.

Antibacterial activity of these newly isolated LAB strains was tested against *C. perfringens* DSM756 as well as six other *Clostridium* strains isolated from sick chickens. *L. salivarius* ICVB421 and ICVB430 strains presented the upmost zones of inhibition, with diameters of 2.07 and 1.63 cm, respectively. *L. reuteri* ICVB416, *L. rhamnosus* ATCC 7469 and *E. faecalis* 14 exhibited less activity against the aforementioned target bacteria, with diameters of inhibition of 1.13, 1.07 and 0.93 cm, respectively (Tables S3 and S4). In this assessment, we noted the peculiar sensitivity of *C. perfringens* ICVB081 to *L. salivarius* strains (Figure 1). 

After neutralizing the pH of the culture-supernatant, the newly isolated *Lactobacillaceae* strains did not display any inhibitory activity, in contrast to the neutralized culture-supernatant of *E. faecalis* 14 used as the positive control (data not shown). These data suggest that antibacterial activity of the newly isolated LAB-strains was attributable to organic acids such as lactic acid, the amount of which ranged from 11.3 to 13.02 g/L for *L. salivarius* strains and 6.91 g/L for the *L. reuteri* strain (Table S5). 

### 3.3. Biofilm Formation

These newly isolated *Lactobacillaceae* strains have various degrees of adhesion to polystyrene. The most efficient strains in terms of biofilm formation were *L. salivarius* ICVB430 and ICVB421, with absorbencies of 4.00 and 2.17 at 600 nm, whereas *L. reuteri* strain ICVB416 showed absorbance of 1.64 (Table S5). 

### 3.4. Resistance to Chicken Gastrointestinal Conditions 

**Figure 2:**
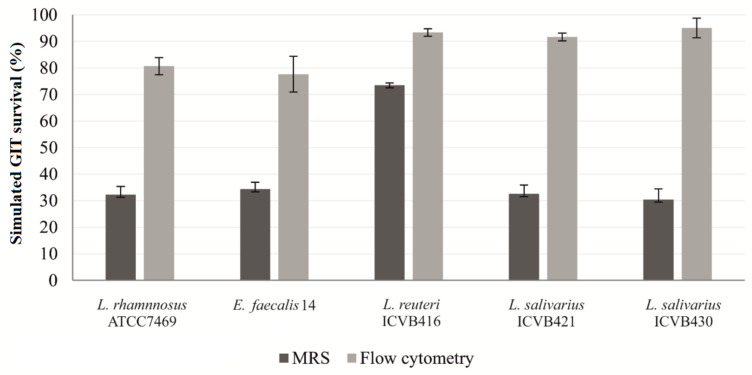
Survival rates of LAB strains under conditions mimicking chicken gastrointestinal environment (%) using classical counting in MRS and flow cytometry. The cytometry assay was performed after labelling the cells with syto-24 and propidium iodide.

Assessment of conditions mimicking the GIT environment made it possible to select strains with a high probability of reaching the intestine. After counting colonies on MRS agar, *L. reuteri* ICVB416 presented a significantly higher level of survival rate to the GIT conditions (73.47%) than other strains, which have survival rates of approximately 32% (Table S5). Nevertheless, when the living cells were quantified using the flow cytometry method, the values augmented as viable but non-culturable (VBNC) cells were included in the cells counts. Interestingly, the newly selected LAB strains possessed better survival rates than the reference strains used, *L. rhamnosus* ATCC7469 and *E. faecalis* 14 (Figure 2). 

### 3.5. Adhesion to Intestinal Cells

**Figure 3:**
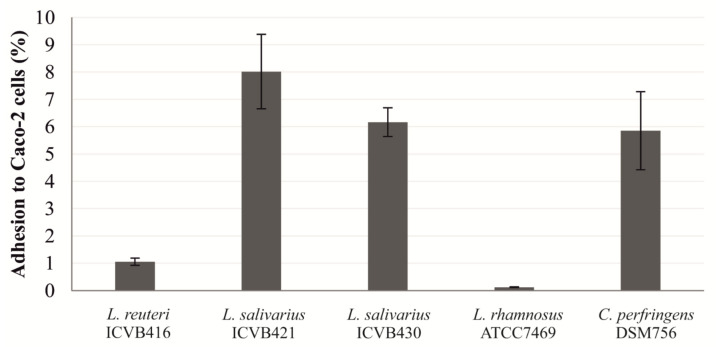
Adhesion percentage of *Lactobacillaceae* strains to eukaryotic Caco-2 cells as determined by quantitative PCR (qPCR).

A real-time qPCR method was used to determine the percentage of adhesion of LAB strains to eukaryotic Caco-2 cells. The percentage of adhesion was calculated in relation to the number of bacteria inoculated. *L. salivarius* ICVB421 has the highest percentage of adhesion (8.02%), more than the pathogenic *C. perfringens* DSM756 strain, which displayed a percentage of 5.85%. It should be noted that, compared to *L. salivarius* strains, *L. reuteri* ICVB416 was weakly adherent with a percentage of adhesion estimated at 1.06%,. Similarly, the reference strain *L. rhamnosus* ATCC7469 displayed a very low level of adhesion (0.19%), as shown in Figure 3. 

### 3.6. Safety Assessment of Lactobacillaceae Strains 

**Figure 4:**
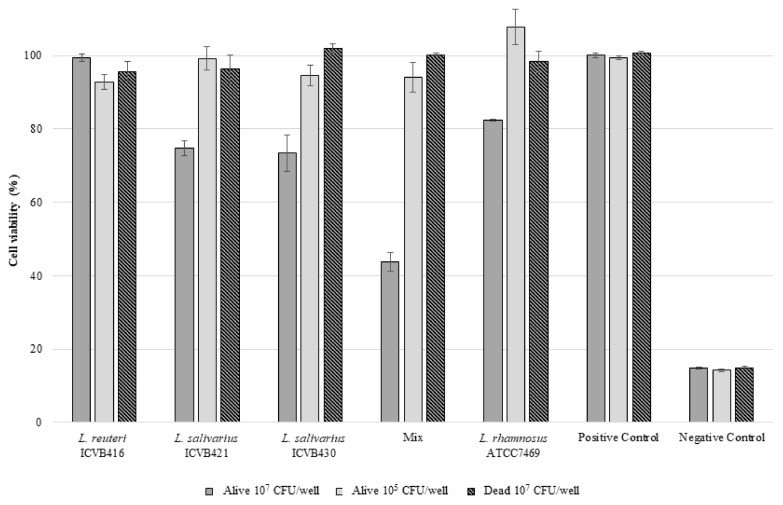
Cell viability (%) of the eukaryotic cell line HT-29 after 24 h contact with LAB. ICVB416: *L. reuteri* ICVB416; ICVB421: *L. salivarius* ICVB421; ICVB430: *L. salivarius* ICVB430. MIX: Mixture in equal volumes of three precedent strains. ATCC7469: *L. rhamnosus* (reference strain). Positive control: Medium without bacteria. Negative control: Triton 0.1%.

None of the *Lactobacillaceae* strains tested here showed hemolytic activity after 24, 48 and 72 h of incubation. Nevertheless, *L. salivarius* ICVB430 and *L. salivarius* ICVB421 exhibited a very limited cytotoxic effect on the eukaryotic HT-29 cell line, with more than 70% viability, but only when they were tested at a concentration of 10^7^ CFU/well. When the three *Lactobacillaceae* strains were tested together, further cytotoxicity was observed decreasing the HT-29 cell viability to less than 40%. When tested at a concentration of 10^5^ CFU/well, no cytotoxic effect on HT-29 cells was registered (Figure 4). Regarding the antibiotic resistance, *L. reuteri* and *L. salivarius* strains exhibited resistance to penicillin G and vancomycin according to critical points established by EUCAST. Notably, resistance to trimethoprim-sulfamethoxazole was observed for *L. salivarius* ICVB430 (Table 1).

### 3.7. Coccidiostat Analysis

The *Lactobacillaceae* strains tested were resistant to diclazuril and sensitive to the rest of the coccidiostat tested. The MIC range was lower than 1 ppm when these *Lactobacillaceae* strains were tested against narasin, salinomycin, maduramicin ammonium and lasalocid A sodium (Table 2). 

### 3.8. In Vivo Trials

#### Zootechnical Performance during the Start-Up Phase (0–14 Days)

**Figure 5:**
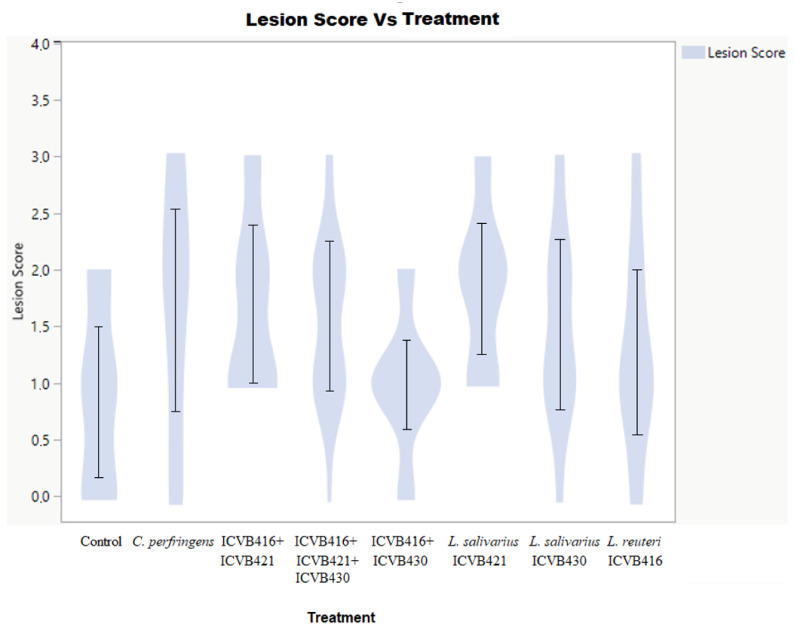
Lesion score layout. It shows the distribution of lesions depending on the treatment. It shows proportionally the number of animals that presented each type of lesion score depending on the treatment.

**Figure 6:**
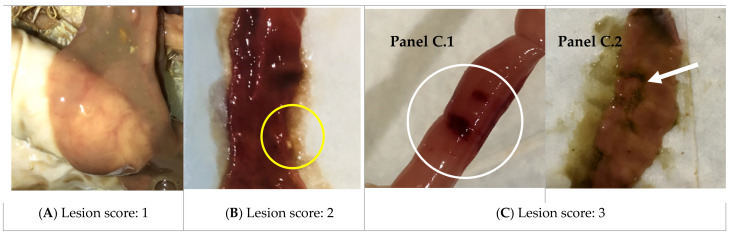
Necrotic enteritis lesions. (**A**) Score 1: intestine is thin and friable; (**B**) Score 2: the picture shows the thin, friable intestine with a small patch of necrosis (yellow circle). There is also redness in the intestinal tract, and some gray mucus is characteristic; (**C**) Score 3: the intestine shows a larger lesion visible through the outer wall of the intestinal tract. There are larger patches of necrosis (white circle and arrow), which are the green areas stained by bile. Panel C.1 shows the closed tube and panel C.2 shows the open tube and exposed inner side above.

The administration of *Lactobacillaceae* strains alone or in mixture in the first two days of the bird’s life did not significantly influence the weight or feed intake of the chickens (Table 3). On day 10, the group treated with the combination of *L. reuteri* ICVB416 and *L. salivarius* ICVB430 presented a significantly higher weight than the control. From days 10 to 14, no significant weight gain or increase in food consumption were observed in the *Lactobacillaceae*-treated groups. Different effects were found from the evaluations of the lesion scores. Interestingly, the group treated concomitantly with *L. reuteri* ICVB416 and *L. salivarius* ICVB430 showed lower lesion scores in comparison to other infected chicken groups (Figure 5 and Figure 6).

## 4. Discussion

Microbial infections such as NE can lead to critical damage and a significant number of deaths when no treatments are applied [55,56,57]. NE is known to afflict the GIT of poultry and cause major economic losses around the world, which can reach up to 6 billion US $ per year [58]. Administration of antibiotics such as penicillin G, amoxicillin, ampicillin, bacitracin, neomycin and tylosin have been suggested as means of preventing NE [59], but strains of *C. perfringens* with resistant phenotypes have been reported [60,61], which delineate the potential of this etiologic agent to defy aging antibiotics. Further measures such as vaccination, bacteriophages, usage of AMPs, prebiotics and probiotics have been insistently proposed to control the bacterial NE infection [62,63]. 

*Lactobacillaceae* strains isolated in this work include *L. reuteri* and *L. salivarius*, which are particularly active against *C. perfringens* through their production of lactic acid as assessed *in vitro*. Although this inhibitory activity is exerted in a strain-dependent manner, the data obtained here are in agreement with those formerly reported [64,65,66]. Lactic acid causes damages in the cell membrane, leading to a cascade of deleterious effects such as inhibition of enzymatic activities, alteration of DNA structure and cell death [67,68]. 

Besides, LAB strain can form biofilms which can be a strategy to control growth of pathogenic bacteria. The formation of biofilms is noticeably important for selecting and designing probiotic candidate strains; even this criterion remains versatile within the LAB group [36]. It is worth noting that the biofilm formation was reported for LAB strains as a means of controlling pathogens such as *Listeria* and *Salmonella* [69,70]. The adhesion of LAB to intestinal cells is considered an element in selecting and characterizing strains, which are candidates for probiotic claims. LAB-strains with such adhesion ability can stand as a barrier against enteropathogens, offering advantages to the host by increasing their time of transit in the gut, helping them compete for nutrients, discarding pathogens from binding sites and increasing the host’s immunity [71,72,73]. In this study, we show that strains of *L. salivarius* are more adherent to Caco-2 cells than those of *L. reuteri*. The scores of adhesion obtained for these newly isolated LAB strains are globally in strong agreement with those reported in the literature [64,74]. The ability of bacteria to defy and survive under the GIT conditions can be simulated *in vitro*, and the resulting data are crucial for validating their suitability for probiotic applications. Interestingly, *L. reuteri* showed a better potential capability to survive the passage through the simulated chicken GIT than other LAB strains tested under similar conditions. This peculiar advantage can be linked to its ability to precipitate the bile salts via the action of bile salt hydrolase [75,76,77]. 

The method of assessing GIT survival by agar enumeration is deemed tedious and does not take into account the VBNC [78]. The survival of LAB strains was confirmed by the flow cytometry method, which includes both viable bacteria and VBNC. As expected, the survival percentages obtained by this method were significantly elevated, compared to data obtained after their growth and enumeration on the MRS media. These observations were remarkably obtained and validated for all strains, and scores were comprised between 80 to 90%. 

It is worth noting that all of the selected *Lactobacillaceae* strains were devoid of hemolytic activity, and assessment of their cytotoxicity on eukaryotic cells revealed a very limited effect of *L. salivarius* strains on HT-29 cells at a concentration of 10^7^ CFU/well, or in conjunction with other selected *Lactobacillaceae* strains. Nevertheless, at 10^5^ CFU/well, this cytotoxic effect was abolished, underlining the importance of cell concentrations, as has been previously reported by Er et al. [79]. The three selected *Lactobacillacae* strains exhibited resistance to some antibiotics, but such a feature is not deemed restrictive for their application [80]. 

Genome analyses of the LAB strains isolated here enabled us to locate gene coding for resistance to antibiotics on chromosomes and permitted us to identify the coding for efflux pumps, which are known to use the energy of ion or substrate-product gradients to expel cytotoxic compounds [81]. On the other hand, resistance to vancomycin is believed to naturally occur in *Limosilactobacillus* and *Ligilactobacillus* species [82], and the genome analyses carried out in this study did not show any genetic mobile element. Taken together, these *in vitro* tests made it possible to design the most promising candidates for probiotic applications. 

Our focus was concentrated on the use of these newly isolated strains as means to control the bacterial NE, which continues to emerge in the poultry livestock as a harmful bacterial infection with large economic consequences. To confirm and strengthen all of the data obtained *in vitro*, additional trials were undertaken to provide evidence of *in vivo* efficacy. To that end, coccidiostats, which are frequently administered for their action against the *Eimeria* parasite, were used. Nonetheless, the effects of coccidiostats were tested on newly isolated *Lactobacillaceae*, and the data obtained for monensin sodium, diclazuril, salinomycin, lasalocid A and robenidine were in strong agreement with previously reported data [83,84]. These newly isolated *Lactobacillaceae* strains were sensitive to narasin, with values of 0.016 to 0.125 ppm; below are those reported for *L. fermentum* strains [85]. 

The administration of *Lactobacillaceae* strains, alone or in conjunction during the first two days of the chicken’s life, did not influence their weights or feed consumption. Only the group treated with *L. reuteri* ICVB416 + *L. salivarius* ICVB430 showed a weight gain, compared to the control on day 10. It is reported that probiotics are most apparent in the first days of life, when the initial microbiota is in development [86]. The effectiveness of probiotics is noticeable after a change in the diet, following any stress disturbance or antibiotic uptake [87]. Moreover, administration of high concentrations of *L. salivarius* (10^9^ CFU/g) impact the microbiota by translating them into an increase in body weight [88]. Other studies have suggested that no significant effects are registered on animal performance when treated with *Lactobacillaceae* probiotics [89,90]. 

The results obtained after infection with *C. perfringens* revealed the advantages of the combination of *L. reuteri* ICVB416 and *L. salivarius* ICVB430, since a significant effect on the weight gain and lesion score of chickens was registered. The synergistic effect between two, or even more, probiotic strains has already been reported. A previous study by Mappley et al. [91] mentioned the benefits of the combination of *L. reuteri* LM1 and *L. salivarius* LM2 against avian intestinal spirochetosis [91]. In line with this, Carter et al. [92] reported a protective effect when *L. salivarius* 59 and *E. faecium* PXN33 were used in combination to reduce *Salmonella* loads in the chicken. Different studies pointed out the protective role of *Lactobacillaceae* strains against *C. perfringens* [93,94,95]. Notable, *Lactobacillaceae* probiotics have been proposed as preventive treatments for the bacterial NE. This is the case for *L. johnsonii* FI9785, which was shown to reduce the extent of colonization and persistence of *C. perfringens* in 20-day-old chicks when it was administered at 10^9^ CFU [96]. On the other hand, *L. fermentum* 1.2029 brought about a reduction of lesions due to NE in chickens by modulating the immune response of the intestinal mucosa [97].

## 5. Conclusions

The combination of *L. reuteri* ICVB416 and *L. salivarius* ICVB430 has a good *in vitro* probiotic capacity, which is translated into an increased performance and preventative treatment against the bacterial NE in chickens. Additional experiments are requested to determine when and how these newly isolated *Lactobacillus* strains are expected to be administered during the chicken’s life.

## Figures and Tables

**Table 1:** Antibiograms of *Lactobacillaceae* strains tested *in vivo*. The sizes of inhibition zones are indicated in mm between brackets.

Antibiotics	Penicillin	Cefotaxime	Gentamicin	Vancomycin	Clindamycin	Ciprofloxacin	Tetracycline	Trimethoprim Sulfamethoxazole
*L. reuteri* ICVB416	R (0)	S (45)	S (26)	R (0)	S (40)	S (36)	S (26)	S (21)
*L. salivarius* ICVB421	R (0)	S (42)	S (28)	R (0)	S (46)	S (26)	S (26)	S (34)
*L. salivarius* ICVB430	R (0)	S (42)	S (28)	R (0)	S (34)	S (26)	S (26)	R (0)

Legend. S: Susceptible, Increased Exposure (A microorganism is classified as increased exposure and susceptible when there is a high probability of therapeutic success because exposure to the agent is increased by adjusting the dosing regimen or by its concentration at the site of infection) R: Resistant (A microorganism is classified as resistant when there is a high probability of therapeutic failure even with increased exposure) (http://www.eucast.org/clinical_breakpoints/, accessed on 3 May 2021).

**Table 2:** Minimal inhibitory concentration (MIC) of coccidiostat usually used in poultry production. Values are expressed in ppm.

Active Agent	Dosage in Farms	MIC		
		*L. reuteri* ICVB416	*L. salivarius* ICVB421	*L. salivarius* ICVB430
Monensin sodium	60–125	2–4	1–2	2–4
Narasin	60–70	0.063–0.125	0.016–0.063	0.016
Salinomycin	30–70	0.5–1	0.125–0.25	0.25–0.5
Maduramicin ammonium	5	0.5–1	0.25	0.25–0.5
Lasalocid A sodium	75–125	0.25–0.5	1–2	0.125–0.5
Diclazuril	1	>512	>512	>512
Robenidine	30–36	4–16	8–16	4–16

**Table 3:** Effects on chicken weight, feed consumption and FCR (Feed Conversion Ratio) after *Lactobacillaceae* supplementation. FCR is the ratio of measuring the efficiency with which the bodies of livestock convert animal feed into the desired output.

	Average Weight/Bird (g)		Feed Consumption (g)		FCR	
	D10	D14	D0-D10	D10-D14	D0-D10	D10-D14
Control (No *L.* addition)	320.4 ± 12.8 ^B^	547.4 ± 22.2 ^AB^	311.50 ± 9.9 ^AB^	280.21 ± 10.3	1.12 ± 0.02 ^AB^	1.24 ± 0.04 ^AB^
*L. reuteri* ICVB 416	317.9 ± 10.7 ^B^	544.8 ± 24.8 ^AB^	305,50 ± 12,3 ^AB^	275.63 ± 12.3	1.12 ± 0.02 ^AB^	1.22 ± 0.05 ^B^
*L. salivarius* ICVB 421	329.8 ± 8.4 ^AB^	564.6 ± 17.9 ^A^	314.17 ± 10.1 ^AB^	285.42 ± 9.0	1.13 ± 0.01 ^AB^	1.22 ± 0.04 ^B^
*L. salivarius* ICVB 430	326.9 ± 17.4 ^AB^	555.4 ± 22.9 ^AB^	316.33 ± 14.7 ^A^	282.08 ± 12.6	1.13 ± 0.02 ^A^	1.23 ± 0.04 ^AB^
ICVB 416 + ICVB 421	320.2 ± 9.2 ^AB^	543.4 ± 20.1 ^AB^	303.17 ± 9.4 ^B^	273.75 ± 7.8	1.11 ± 0.03 ^AB^	1.23 ± 0.02 ^AB^
ICVB 416 + ICVB 430	334.1 ± 15.06 ^A^	566.5 ± 20.0 ^A^	314.67 ± 9.1 ^AB^	284.38 ± 9.8	1.10 ± 0.03 ^B^	1.22 ± 0.04 ^AB^
ICVB 416 + ICVB 421 + ICVB 430	320.9 ± 12.3 ^AB^	536.7 ± 16.0 ^B^	304.79 ± 8.4 ^AB^	274.58 ± 8.7	1.13 ± 0.04 ^AB^	1.27 ± 0.05 ^A^

Statistical significances between groups are represented by different letters (A, B) that mean *p* < 0.05.

## Data Availability

Raw data will be provided upon request.

## Supplementary Materials

The following supporting information can be downloaded at: https://www.mdpi.com/article/10.3390/microorganisms10010152/s1, Table S1: Feed formula, Table S2: Nutrient composition, Table S3: Anti-*Clostridium perfringens* DSM756 activities of lactic acid bacteria, Table S4: Anti-*Clostridium perfringens* ANSES Clin1 activities of lactic acid bacteria, Table S5: Characterization of LAB from the study.
