# Supplementary material for: Protective Effects of Novel Lactobacillaceae Strains Isolated from Chicken Caeca against Necrotic Enteritis Infection: In Vitro and In Vivo Evidences
Source: Microorganisms. 2022 Jan 12;10(1):152. doi: 10.3390/microorganisms10010152 (PMC8780607; doi:10.3390/microorganisms10010152)
Supplement: Supplementary file 1 [file microorganisms-10-00152-s001.zip › microorganisms-1528067-supplementary.pdf]

microorganisms

Supplementary Data

Table S1. Feed formula.

.	Starter diet (0-9 days)	Grower diet (10-17 days)
Corn	60.83	34,87
SBM (48%)	33.91	28,14
Wheat	-	20,00
Dried distillation grains	-	10,00
Fat	1.17	3,06
Limestone	1.47	1,45
Monocalcium phosphate	1.54	1,44
NaCl	0.44	0,28
L-Lysine HCl	0.15	0,26
DL-Methionine	0.21	0,21
L-Threonine	0.06	0,08
Vitamin premix	0.18	0,18
Mineral premix	0.05	0,05

Table S2. Nutrient composition.

	Starter diet (0-9 days)	Grower diet (10-17 days)
Dry matter	87.98	88.93
Protein, crude	22.00	22.00
Fat, crude	3.63	5.87
Fiber, crude	2.,17	2.89
Ash	5.45	5.54
Lysine	1.3	1.3
Calcium	0.88	0.88
Phosphorus, total	0.70	0.71
Apparent Metabolizable Energy (AME) (Kcal/kg)	3 000,00	3 000,00

Table S3. Anti-*Clostridium perfringens* DSM756 activities of lactic acid bacteria.

**Level of activity**	**Specie**	**Number of strains**	**Strains**
-	*L. reuteri*	2	ICVB413, 3DMV2
	L. salivarius	0	-
	Other	0	-
+	*L. reuteri*	1	ICVB493
	L. salivarius	0	-
	Other	1	*L. gasseri* SE.3
++	*L. reuteri*	11	2GE8, ICVB412, ICVB414, S5.5, ICVB415, S5.12, ICVB416, ICVB417, ICVB418, ICVB419, 2GMV9
	L. salivarius	27	ICVB420, 3GRM9, ICVB421, ICVB422, 2GRM1, 2GE1, ICVB424, ICVB425, ICVB426, ICVB427, ICVB428, ICVB429, ICVB430, ICVB431, ICVB432, S4.15, ICVB433, ICVB434, ICVB435, ICVB436, 2GRM6, 2GMV1, ICVB437, S3.15, ICVB438, ICVB439, ICVB440
	Other	6	*L. gallinarum* SE.2, *St. alactolyticus* SE.1 *Strep. alactolyticus* 3DE6, *Strep. lutetiensis* S4.18, *L. johnsonii* SE.4 et *L. antri* ICVB441.
+++	*L. reuteri*	0	-
	L. salivarius	1	ICVB423
	Other	0	-

Legend. - (+ : no halo, + : halo size between 0,2-0,4 cm; ++ between 0,4 – 0,8 cm; and +++ between 0,8 - 1,4 cm).

Table S4. Anti-*Clostridium perfringens* ANSES Clin1 activities of lactic acid bacteria.

Level of activity	Specie	Number of strains	Strains
-	*L. reuteri*	1	3DMV2
	L. salivarius	0	-
	Other	0	-
+	*L. reuteri*	6	2GE8, ICVB413, ICVB414, ICVB418, ICVB419, 3GMV7
	L. salivarius	8	ICVB422, 2GRM1, ICVB425, ICVB426, ICVB427, ICVB429, ICVB436, 2GMV1
	Other	7	*Lb. gasseri* SE.3, *Lb. gallinarum* SE.2*, St. alactolyticus* SE.1 *St. alactolyticus* 3DE6*, St. lutetiensis* S4.18*, Lb. johnsonii* SE.4 et *Lb. antri* ICVB441
++	*L. reuteri*	6	S5.5, ICVB415, S5.12, ICVB416, ICVB417, ICVB493
	L. salivarius	20	ICVB420, 3GRM9, ICVB421, 2GE1, ICVB423, ICVB424, ICVB428, ICVB430, ICVB431, ICVB432, S4.15, ICVB433, ICVB434, ICVB435, 2GRM6, ICVB437, S3.15, ICVB438, ICVB439, ICVB440
	Other	0	-
+++	*L. reuteri*	1	ICVB412
	L. salivarius	0	-
	Other	0	-

Legend. - (+ : no halo, + : halo diameter size between 0,2-0,4 cm; ++ between 0,4 – 0,8 cm; and +++ between 0,8 - 1,4 cm).

Table S5. Characterization of LAB from the study.

Specie	Strain	Lactic acid production (g/L)[1]	Biofilm formation (OD600)[2]	Survival GIT conditions[3]
L. reuteri	ICVB412	7,63 ± 1,03	2,19 ± 0,11	90,27 ± 0,38
	ICVB417	8,16 ± 0,41	1,67 ± 0,08	84,95 ± 2,16
	ICVB416	6,91 ± 0,61	1,64 ± 0,18	93,34 ± 1,41
	S5.12	5,39 ± 0,30	1,14 ± 0,09	-
	ICVB413	5,49 ± 0,31	0,99 ± 0,07	83,88 ± 1,00
	ICVB414	5,32 ± 0,26	0,95 ± 0,06	78,06 ± 3,00
	ICVB493	9,47 ± 0,31	0,83 ± 0,06	85,25 ± 1,49
	3DMV2	7,62 ± 0,49	0,80 ± 0,07	-
	2GE8	7,26 ± 0,72	0,68 ± 0,07	-
	2GMV9	9,79 ± 0,51	0,37 ± 0,03	-
	ICVB418	12,48 ± 2,10	0,37 ± 0,10	84,06 ± 1,37
	S5.5	6,68 ± 0,62	0,31 ± 0,04	-
	ICVB415	10,00 ± 0,38	0,31 ± 0,03	90,23 ± 0,39
	ICVB419	13,71 ± 0,98	0,27 ± 0,03	81,92 ± 1,80
L. salivarius	ICVB430	13,02 ± 2,76	4,00 ± 0,01	95,05 ± 3,73
	ICVB423	15,42 ± 1,43	3,38 ± 0,39	86,12 ± 0,41
	ICVB421	11,30 ± 2,02	2,17 ± 0,20	91,67 ± 1,45
	ICVB433	7,41 ± 0,38	1,12 ± 0,07	-
	ICVB429	13,39 ± 0,42	0,96 ± 0,20	79,58 ± 1,32
	ICVB437	10,11 ± 1,44	0,90 ± 0,14	-
	ICVB422	9,82 ± 0,75	0,83 ± 0,06	-
	ICVB424	11,55 ± 0,33	0,77 ± 0,07	-
	2GRM1	11,25 ± 3,32	0,77 ± 0,21	-
	ICVB435	9,85 ± 2,24	0,77 ± 0,35	-
	3GRM9	11,20 ± 2,13	0,73 ± 0,07	-
	ICVB431	12,88 ± 1,31	0,71 ± 0,07	-
	ICVB428	7,71 ± 0,57	0,66 ± 0,04	-
	2GRM6	11,17 ± 1,65	0,66 ± 0,10	-
	2GMV1	12,23 ± 1,07	0,66 ± 0,09	-
	S4.15	10,18 ± 0,85	0,66 ± 0,07	-
	ICVB434	11,85 ± 2,97	0,65 ± 0,15	-
	ICVB420	9,20 ± 2,10	0,64 ± 0,04	-
	S3.15	8,74 ± 0,86	0,62 ± 0,16	-
	ICVB427	8,64 ± 2,16	0,61 ± 0,12	-
	ICVB432	9,62 ± 0,10	0,56 ± 0,03	-
	ICVB440	12,87 ± 0,36	0,44 ± 0,04	-
	ICVB438	8,91 ± 1,70	0,44 ± 0,04	-
	ICVB439	13,00 ± 0,85	0,41 ± 0,02	-
	ICVB426	8,26 ± 0,87	0,36 ± 0,07	-
	ICVB436	13,90 ± 0,86	0,35 ± 0,05	-
	2GE1	12,86 ± 2,11	0,27 ± 0,03	-
	ICVB425	10,10 ± 0,91	0,20 ± 0,01	-
L. antri	ICVB441	6,99 ± 0,70	1,11 ± 0,38	-
L. johnsonii	SE 4	9,67 ± 1,38	0,31 ± 0,02	-
L. gallinarum	SE.2	7,58 ± 0,86	0,61 ± 0,14	-
L. gasseri	SE.3	6,91 ± 1,24	0,37 ± 0,09	-
Strep. alactolyticus	3DE6	8,16 ± 2,01	0,93 ± 0,20	-
Strep. alactolyticus	SE 1	8,35 ± 1,07	0,38 ± 0,03	-
Strep. lutetiensis	S4 18	4,65 ± 0,70	0,42 ± 0,01	-
L. rhamnosus	ATCC 7469	-	-	80,68 ± 3,23
Ent. Faecalis	DD14	-	-	77,62 ± 6,77

[1]Lactic acid production (g/L) after incubation for 20 h in MRS medium at 37ºC. Quantification by HPLC using the isocratic method (Eluent: 0.05% H_3PO_4). [2]Biofilm formation of lactic acid bacteria. Measurement by spectrometry at 600nm (OD_{600nm}). [3]Survival to chicken gastrointestinal conditions (%).
